# Nitrogen–Salt Interaction Adjusts Root Development and Ion Accumulation of the Halophyte *Suaeda salsa*

**DOI:** 10.3390/plants11070955

**Published:** 2022-03-31

**Authors:** Shoule Wang, Shaoqing Ge, Changyan Tian, Wenxuan Mai

**Affiliations:** 1 State Key Laboratory of Desert and Oasis Ecology, Xinjiang Institute of Ecology and Geography Chinese Academy of Sciences, Urumqi 830011, China; wangshoule17@mails.ucas.ac.cn (S.W.); geshaoqing18@mails.ucas.ac.cn (S.G.); 2 University of Chinese Academy of Sciences, Beijing 100049, China

**Keywords:** nitrogen, root distribution, salinity, *Suaeda salsa*, interaction

## Abstract

Nitrogen (N) application might exert a great impact on root (biomass, length) distribution, which possibly contributes to ion and nutrient uptakes. Here, we address the effects of N application on these characteristics to detect how N improves its salt tolerance. *Suaeda salsa* was subjected to four salt levels (0.5, 1.0, 1.5, and 2.0%) and three N treatments (${\mathrm{NO}}_{3}^{-}$-N: 0, 0.25, and 0.50 g·kg^−1^) in soil column experiments. The N applications performed a “dose effect” that significantly enhanced the growth of Suaeda at low salt levels, while negative effects were displayed at high salt levels. Moderate N markedly benefited from ${\mathrm{Na}}^{+}$ and ${\mathrm{Cl}}^{-}$ uptake, which was approximately 111 mg and 146 mg per plant at a salt level of 1.0%. Exposure to a certain N application significantly enhanced topsoil root length at salt levels of 0.5% and 1.0%, and it was higher by 0.766 m and 1.256 m under N50 treatment than that under N0 treatment, whereas the higher salt levels accelerate subsoil root growth regardless of N treatment. Therefore, its interactive effects on root development and ion uptake were present, which would provide further theoretical basis for improving saline soil amelioration by N application. Regression analysis always showed that topsoil root length generated more positive and significant influences on ion uptake and vegetative growth than total root length. The results suggested that N application is beneficial to salt tolerance by altering root allocation so as to raise its elongation and gather more ions for halophyte in the topsoil.

## 1. Introduction

Salt injury is a serious threat to agriculture worldwide, and phytoremediation is a feasible and effective method to rescue saline-alkali land [1,2,3]. Halophytes can uptake soil salt and effectively improve the quality of saline-alkali land [4,5,6]. In addition to the effects of salt stress, halophyte growth may be inhibited by nutrient deficiencies in saline soil. Nitrogen (N) may improve the synthesis, transport, and storage of plant photosynthates, which are activities conducive to various plant life processes [7,8,9]. The presence of N not only significantly increases its accumulation in halophytes, but also increases in osmotic substances, such as proline, soluble sugar, and ${\mathrm{Na}}^{+}$, which enhance salt resistance [10,11]. Benzarti et al. [12] found that N mitigates the negative impacts of salinity on plant growth by significantly increasing photosynthetic activity parameters and the photosynthetic N-use efficiency. Song et al. [13] revealed that appropriate ${\mathrm{NO}}_{3}^{-}$-N application plays an important osmotic role in *Suaeda salsa* under high-salinity conditions, whereas it markedly decreases the shoot ${\mathrm{Cl}}^{-}$ content and has no effect on seedling emergence. It should be noted that plant ion uptake processes a closer link with N efficiency [14]. A thorough understanding of the salt-uptake characteristics of halophytes after N addition is important for improving N-use efficiencies of plants growing on saline-alkali land.

The root directly takes up salt and nutrients, and its morphology and configuration are important for halophyte responses to environmental stress [13,15,16]. Generally, the root morphology and architecture depend on N efficiency to a certain extent. This distribution pattern also affects the root distribution in the soil [17,18]. The presence of N can effectively improve root formation and enhance both taproot development and lateral root elongation [19,20]. Chen et al. [21] deemed that a certain level of N being present would accelerate cotton lateral root elongation and that of the root-projected area by regulating abscisic acid and salicylic acid. Base ions and N in soil, however, are highly mobile and show uneven spatial distributions with obvious spatiotemporal variations [22,23]. To adapt to the non-homogeneity of nutrients and salt, root growth might show a strong plasticity response [24]. Moderate salt levels displayed a positive effect on root development for halophyte, but the stress from higher salt levels causes a negative effect, where it obtains more nutrients by increasing fine root presence in the subsoil [25]. N application would be bound to alter nutrient distributions in the topsoil and subsoil, which might produce an important influence on root architecture. For instance, Drescher et al. [26] found that N availability in a soil profile vertically influences root morphology and total nutrient uptake, and soils with deep, available N levels likely allow plants to optimally supply photosynthates. Nevertheless, it was not explicitly mentioned that more ion, along with water, reaches the topsoil due to evaporation in saline soil, and whether halophyte roots perform similarly under a N supply level is still unclear. Additionally, it is unclear what the relationship is between root distribution and ion uptake under N application, and what the impacts of salt on root elongation and ion (${\mathrm{Na}}^{+}$, ${\mathrm{Cl}}^{-}$) accumulation are. Clarifying these issues would facilitate further understanding of the mechanism in intensifying salt tolerance of halophyte under N application.

*Suaeda salsa* is a euhalophyte with a high tolerance to salt stress owing to both its morphology and physiology, and it can grow well in 400 mM NaCl [6,27]. It is an important model plant for studying saline–alkali soil improvements related to the vacuolar localization’s role in gathering more ${\mathrm{Na}}^{+}$ and leaf succulence under a decreasing osmotic potential [6,28,29]. Therefore, this study used *Suaeda salsa* to analyze the growth, root distribution, and ion and nutrient uptake responses to N applications in a pot experiment. The aims of this study were to: (1) clarify the effects of N application on root distribution under salt-stress conditions; (2) investigate the differences in nutrient- and ion- absorption of *Suaeda salsa* receiving different N treatments; and (3) explore the potential relationship between root development and ion uptake.

## 2. Results

### 2.1. Plant Biomass and Root Length

**Figure 1:**
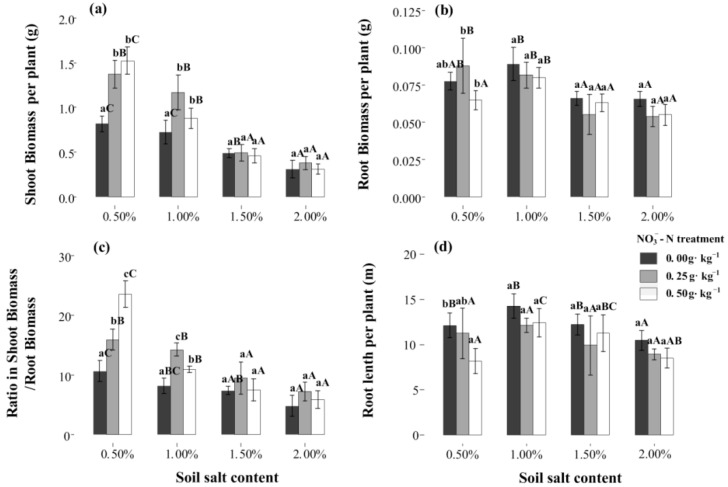
Effects of ${\mathrm{NO}}_{3}^{-}$-N treatments on *Suaeda salsa* grown at different salt levels. (**a**) Shoot biomass, (**b**) root biomass, (**c**) shoot/root ratio, and (**d**) root length. Capital letters indicate significant differences among the four salt levels. Lowercase letters indicate significant differences among the three ${\mathrm{NO}}_{3}^{-}$ -N treatments.

At the S1 salt level, N application significantly enhanced the shoot biomass, and it exceeded to 1.52 g per plant under N50 treatment (*p* < 0.05). However, at higher salt levels (S3, S4), the positive and promoting effect only occurred under N25 treatment (Figure 1a). The response of root biomass and length to salt and N application was obviously different from the response of shoot biomass. At higher salt levels, the root biomass displayed a negative response to N application (Figure 1b). Under the same N treatment, the root biomass was highest at salt level S2, where it reached 0.079–0.89 g per plant. Similar to shoot biomass, a certain N application significantly accelerates the shoot/root ratio (*p* < 0.05, Figure 1c). Additionally, N application always presented some negative effects on root length, which was decreased by 7.8–32.6% under the N50 treatment (Figure 1d).

### 2.2. Distributions in Root Biomass and Root Length

**Figure 2:**
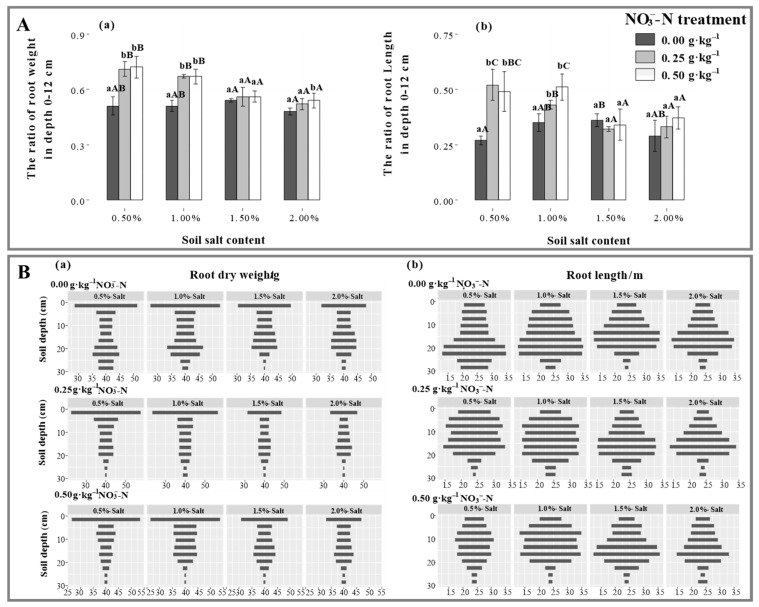
Effects of ${\mathrm{NO}}_{3}^{-}$-N treatments on the root biomass and length distribution of *Suaeda salsa* grown at different salt levels. (**A**(**a**,**b**)) represents the ratio of root weight to length in depth 0–12 cm. (**B**(**a**,**b**)) represents the root biomass and length distribution. Capital letters indicate significant differences among the four salt levels. Lowercase letters indicate significant differences among the three ${\mathrm{NO}}_{3}^{-}$ N treatments.

At lower salt levels (S1, S2), N application gradually increased the root biomass in depth 0–3 cm (*p* < 0.05), which was 55.5% and 31.6% higher under the N25 treatment than under the N50 treatment, whereas it decreased by 4.1% and 24.2% when in depth 4–30 cm. At higher salt levels (S3, S4), however, N25 treatment significantly reduced root production, which was consistent with total root biomass. As a whole, except for root biomass in depth 0–3 cm, it first increased and then decreased under N0 treatment, while the performance vanished under N25 and N50 treatments (Figure 2(Ba)). Additionally, it was found that N application significantly accelerated root growth in depth 0–12 cm (*p* < 0.05), while it disappeared with salinization (Figure 2(Aa)).

N application significantly increased the root lengths of Suaeda at the depth of 0–12 cm at lower salt levels, especially at the S2 salt level, which was 0.24 m and 1.26 m higher under the N25 and N50 treatments than that under N0 treatment (Figure 2(Bb)). No significant and vertical difference in root length by N application under higher salt levels was observed. Under the N25 treatment, salinization enhanced fine root performance in subsoil, in contrast to the N50 treatment, where root length displayed greater at the S2 salt level. N application played a significant and positive role in root elongation on the surface layer (0–12 cm) under lower salt levels (*p* < 0.05), and after N treatments, the root growth performed a significant and negative response to salinization (Figure 2(Ba)).

### 2.3. Nutrient and Na^+^, Cl^−^, K^+^, Salt Uptake by Shoots

**Figure 3:**
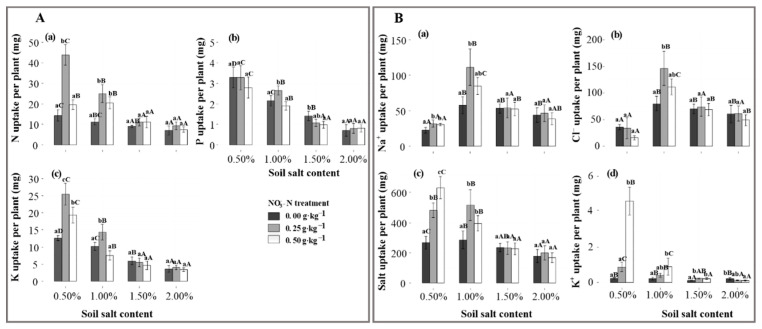
Effects of salt and ${\mathrm{NO}}_{3}^{-}$-N on nutrient (**A**) and ion (**B**) uptake by the shoots of *Suaeda salsa*. (**A**(**a**–**c**)) represent the absorptions in nitrogen (N), phosphorus (P), and potassium (K), and (**B**(**a**–**d**)) represent the absorptions in ${\mathrm{Na}}^{+}$, ${\mathrm{Cl}}^{-}$, salt, and $K^{+}$. Capital letters indicate significant differences among the four salt levels. Lowercase letters indicate significant differences among the three—N treatments.

The highest and most significant uptake in N, P and K was attained in the N25 application, mainly at the S1 and S2 salt levels (*p* < 0.05), while the performance vanished at the S3 and S4 salt levels (Figure 3(Aa–c)). After the N25 application, the nutrient uptake significantly decreased with salinization (*p* < 0.05), which was 34.5, 2.48 and 21.4 g lower under the S4 salt level than that under the S1 salt level. 

N application had significant effects on the ion and salt uptakes of Suaeda. The uptakes of ${\mathrm{Na}}^{+}$, ${\mathrm{Cl}}^{-}$, and salt by Suaeda were greatest under the N25 station, and this was most obvious at the S2 salt level (about 0.111 g, 0.146 g, and 0.516 g per plant) (*p* < 0.05, Figure 3(Ba–c)). At lower salt levels, N application significantly increased $K^{+}$ absorption, but it significantly decreased at the S4 salt level (*p* < 0.05, Figure 3(Bd)). Under the N25 treatment, the ${\mathrm{Na}}^{+}$ and ${\mathrm{Cl}}^{-}$ uptakes were significant and highest at the S2 salt level. 

### 2.4. Relationship of Root Length with Nutrient and Ion Uptake

**Figure 4:**
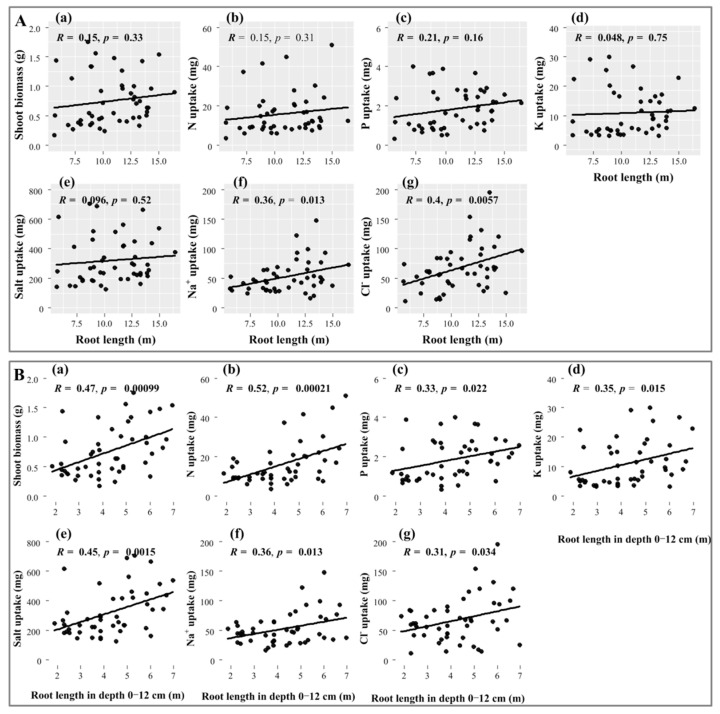
Relationships of total root length (**A**) and root length in depth 0–12 cm (**B**) with shoot biomass, nutrient, and ion uptakes of *Suaeda salsa*. (**Aa**,**Ba**), shoot biomass; (**Ab**,**Bb**), N uptake; (**Ac**,**Bc**), K uptake; (**Ad**,**Bd**), P uptake; (**Ae**,**Be**), salt uptake; (**Af**,**Bf**), ${\mathrm{Na}}^{+}$ uptake; (**A**,**Bg**), ${\mathrm{Cl}}^{-}$ uptake.

Regression analysis showed that in N applications, the total root length of Suaeda was significantly and positively correlated with ${\mathrm{Na}}^{+}$ and ${\mathrm{Cl}}^{-}$ uptake, where R values were 0.36 and 0.4, respectively (*p* < 0.05), but not with N, P, and K uptake (Figure 4A). The significant and higher correlation coefficients with the topsoil root length (in depth 0–12 cm) and with ion uptake and biomass were always displayed, as shown in Figure 4(Bd–f) (*p* < 0.05), and the R values were 0.45, 0.36 and 0.31, respectively. Meanwhile, the topsoil root length produced positive and significant impacts on Suaeda growth and N, P, and K uptake (*p* < 0.05, Figure 4(Ba–d)). 

### 2.5. Interactive Analysis

**Figure 5:**
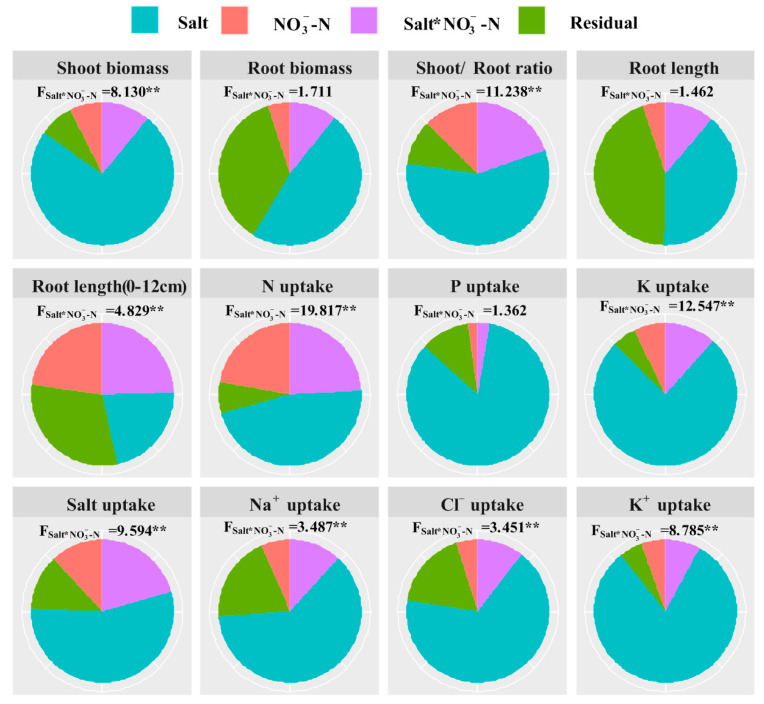
Results (F-value) of two-way ANOVA on the interactive effects of salt and N application on shoot and root biomass, root length and surface area, and ion and nutrient absorption of *Suaeda salsa*. The sector areas represent the ratios of the values in the sum of squares (SS: Salt, ${\mathrm{NO}}_{3}^{-}$-N, Salt *${\;\mathrm{NO}}_{3}^{-}$ -N and Residual) to total sum of square (SST). ** represented the significant level of 0.01.

Compared with N application, salt level displayed a significant and greater influence on the growth and ion and nutrient absorption of Suaeda (Figure 5). Additionally, variances of N application remained a markedly higher level for shoot biomass, root development, and salt absorption (*p* < 0.05). It was found that the interactive effect on root elongation (0–12 cm) was significant, though this was not the case for total root growth (i.e., biomass and length). The significant and interactive effects on ion and salt absorption by salt and N application are presented in Figure 5 (*p* < 0.05).

## 3. Discussion

In this study, ${\mathrm{Na}}^{+}$ was essential for halophyte growth, but a high concentration had a negative influence on saline soil, which was consistent with previous studies [30,31,32]. Additionally, N is a critical factor in the life histories of halophytes [13]. A certain N-treatment level significantly promoted shoot biomass in Suaeda (Figure 1), as was reflected in several previous studies [8,11,13]. ${\mathrm{NO}}_{3}^{-}$-N is also an important signal molecule that stimulates the establishment of root systems [33]. Although Suaeda had a great demand for N, the stimulatory effects of high N treatments on growth weakened under high-salinity conditions. The promotive effects of N application on Suaeda growth showed an obvious “dose effect”, which was closely correlated with the soil-salt level in this study (Figure 1). In low-salinity soil, ${\mathrm{NO}}_{3}^{-}$-N treatments resulted in apparent and positive shoot biomass responses, but this effect diminished in high-salinity soil (exceeding the 1.0% level). This might be because the positive effect of the ${\mathrm{NO}}_{3}^{-}$N application was not significant along with the increased salinization. The significant reductions in N and P accumulations at the 0.5% salt level after high ${\mathrm{NO}}_{3}^{-}$-N treatments suggested that N absorption differed depending on the available N level. Thus, there was a N-related “dose effect” on Suaeda growth, which was similar to the results of previous studies [7,9]. When the state of N addition is the threshold, the positive effects on the shoots of plants are prominent, and when the threshold is exceeded, the negative effects occur. These results were explained by the interactive analysis. This would provide an insight into how to achieve maximizations in aid to saline soil by phytoremediation.

**Figure 6:**
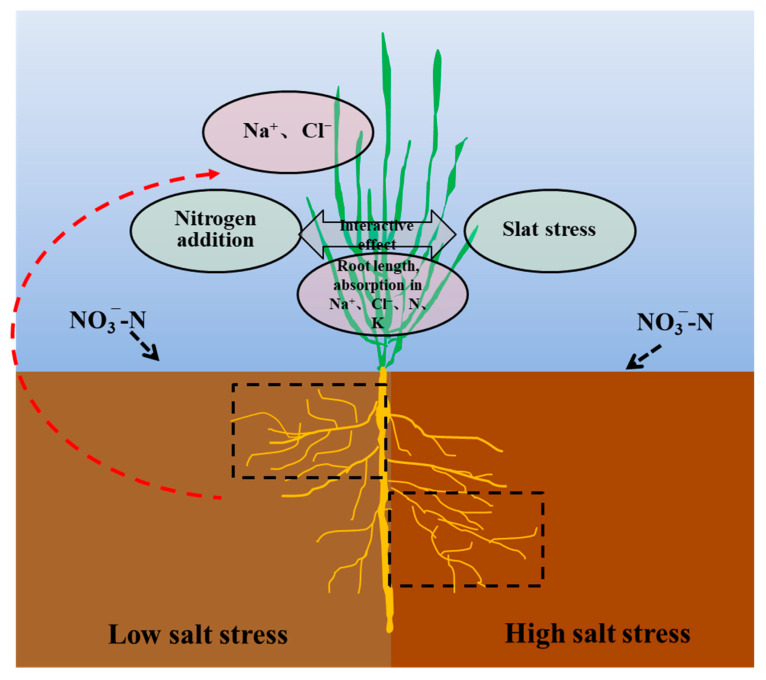
Diagram of the interactive effect between salt and ${\mathrm{NO}}_{3}^{-}$-N on root growth and ion accumulation of *Suaeda salsa*. Dotted boxes represent the positive effect on root elongation in the different soil layer.

The effects of N application on root biomass showed a trend contradictory to that of shoot biomass in this experiment. This result was consistent with the root responses of non-halophytes to N application, in which root biomass decreases and shoot biomass increases, resulting in an increase in the shoot/root ratio [34]. This is more a reflection of the growth strategy; consequently, the biomass distribution was markedly different between the roots and shoots, thereby facilitating the nutrient-use efficiency. Photosynthate allocation is a significant factor in responses to salt stress. The shoot/root ratio was significantly lower at the 2.0% salt level than at the 1.0% salt level, and the ${\mathrm{NO}}_{3}^{-}$-N treatment increased the ratio. This indicated that halophytes possess very fine adjustment actions for root configuration that increase the contact area to allow the uptake of more nutrients and water, which is also an efficient survival mechanism for Suaeda under adverse conditions. Additionally, an appropriate N level benefits halophytes, allowing them to increase their salt-tolerance capabilities. In addition, this study showed that a greater root length occurred at the 1.0% salt level, which was similar to the results of Wang et al. [25]. This suggested that a certain amount of salt promotes the root formation of halophytic plants, whereas levels exceeding the threshold inhibit root formation. The effect pattern of ${\mathrm{NO}}_{3}^{-}$-N on the root distributions in different soil layers changed significantly with the salt levels (Figure 2). The ${\mathrm{NO}}_{3}^{-}$-N supplementation significantly reduced the root lengths and root surface areas of Suaeda in saline soil, mainly because the higher nutrient availability reduced the contact area with the soil. The N25 treatment significantly increased the root length at a depth of 0−12 cm at the S1 and S2 salt levels, which may be because the increased N availability resulted in more fine roots or lateral roots being distributed in the surface soil [35,36]. Under high-salinity conditions, appropriate ${\mathrm{NO}}_{3}^{-}$-N treatments did not increase the root lengths in the surface soil, mainly because more salt was concentrated in the surface soil during transpiration. Although the availability of N in the surface soil was high, the excessive amount of base ions resulted in salt damage to the Suaeda root system. Consequently, the root system reached deeper into the soil, collecting more water and nutrients. Regression analysis demonstrated that the Suaeda growth was closely related to topsoil root length, but not total root length, which suggested that root distribution in the vertical direction emerges as a crucial influence on vegetative growth (Figure 6).

*Suaeda salsa* has a strong ability to assimilate ${\mathrm{Na}}^{+}$ into tissues, and N application has an important stimulating effect on absorption, which occurred in this study (Figure 3(Ba)). It could be proven by the significant interactive effects of nitrogen and salt on ion accumulation (Figure 5). It is considerable that N application enhances the nitrate reductase activity level in the shoots, plays a significant role in organic N production in mesophyll tissues, and increases the nutrient balance under salt-stress conditions [11]. This reinforced the osmotic adjustment capability, which, in turn, allows the plant to effectively absorb more base ions. At the lower salt levels, significant and higher $K^{+}$ content occurred under N50 treatments than that under N0 treatments, which may result from the N addition counterbalancing the increase in K released from soil to accelerate absorption [37]. However, the negative response of $K^{+}$ content to markedly ${\mathrm{NO}}_{3}^{-}$-N treatments was demonstrated at the 2.0% level. Yao et al. [38] found that a N deficiency significantly affects the $K^{+}$/${\mathrm{Na}}^{+}$ ratio in the young leaves, as well as the survival of salt-stressed poplar. Therefore, we concluded that moderate N application is beneficial to ameliorating and utilizing alkaline soil through phytoremediation.

## 4. Materials and Methods

### 4.1. Field Soil Properties

The experiment was conducted in a greenhouse under a 14-h light/10-h dark photoperiod (25 °C day/20 °C night) from June to November, 2019. The soil in this experiment was sampled at a depth of 0–20 cm and was taken from an experimental station in Changji (44°09′59″ N, 87°04′56″ E). The basic physical and chemical properties were as follows: soil pH, 7.64; soil EC, 1.32 ms·cm^−1^; soil salt content, 0.50%; soil available N (${\mathrm{NO}}_{3}^{-}$-N and ${\mathrm{NH}}_{4}^{+}$-N), 33.15 mg·kg^−1^, soil available phosphorus (P), 5.13 mg·kg^−1^; and soil available potassium (K), 280 mg·kg^−1^.

### 4.2. Experimental Design

**Figure 7:**
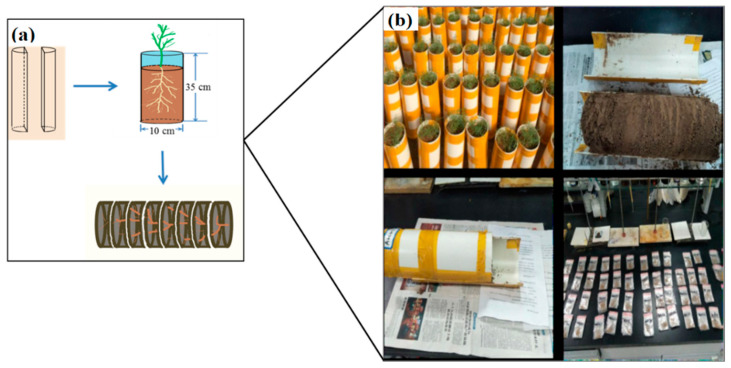
Experimental device and root samples. (**a**) Experimental setup. (**b**) Experimental procedure from seeding to root collection.

The experimental device is shown in Figure 7a. The material used was PVC pipe with an inner diameter of 10 cm and a height of 35 cm. At first, the PVC pipe was divided longitudinally, and then adhesive tape was used in accordance with the original shape. The bottom was sealed with a bottom cover with an inner diameter of 10 cm, which formed a cylindrical container without a mouth. In this experiment, with 48 pots, the salt contents were set at 0.5, 1.0, 1.5, and 2.0% (S1, S2, S3, and S4) and the nitrogen [Ca(NO_3_)_2_] treatments were set at 0, 0.25, and 0.50 g·kg^−1^ (N0, N25, and N50). To investigate the spatial distributions of roots, soil columns were divided into 10 layers, and each layer was 3 cm.

### 4.3. Root Distribution and Ion Uptake

Each pot was filled with 3.1 kg of saline soil. To ensure the nutrient requirements for the growth and development of plants were met, 0.2 g·kg^−1^ Olsen-P was applied (potassium dihydrogen phosphate) to each pot. Then, 20 Suaeda seeds were sown into each pot and allowed to grow to 4 cm before thinning. Salt (NaCl) and N [Ca(NO_3_)_2_] were dissolved into water and added every 3 days, for a total of five times. The gravimetric method was used to supply water to field capacity (20%, *w*/*w*).

After 45 days of growth, the shoots and roots of Suaeda were separated in each pot. The shoots were treated at 105 °C for 30 min and dried at 65 °C for 48 h prior to calculating the biomass. Then, the PVC pipes were opened along the center line, and the soil columns, having depths of 30 cm, were divided into 10 layers (Figure 7b). The roots were collected and washed to remove the adhering soil. The roots were scanned using an Epson V 750 root scanner at a resolution of 400 dpi and analyzed with DT Scan software (Delta-T Devices, Burwell, UK) to obtain root parameters, such as root lengths. Then, the roots were treated at 105 °C for 30 min and dried at 65 °C for 48 h prior to calculating the biomass. Shoot nitrogen (N) and phosphorus (P) were extracted with 18 mol·L^−1^ H_2_SO_4_, and measured with a Kieldahl Azotometer (Kjeltec 8420) and UV spectrophotometer (CARY-60; Bao, 2000). Shoot-diluted extracts were analyzed for ${\mathrm{Na}}^{+}$, $K^{+}$(Flame Photometer, 735 ICP-OES), and ${\mathrm{Cl}}^{-}$ (AgNO_3_ titration method) [39]. 

### 4.4. Statistical Analysis 

We conducted a one-way analysis of variance (ANOVA) to detect the differences in biomass, root length, and ion absorption, and a two-way analysis of variance to analyze the interaction under the different salt and ${\mathrm{NO}}_{3}^{-}$-N treatments using SPSS statistical software (SPSS version 19.0, IBM SPSS Inc., Chicago, IL, USA). Then, the vertical root distributions of Suaeda under different salinity and N treatments were investigated using R software (version 4.0.3-win). Significant differences among means were separated using the LSD (least significant difference) test at the *p* < 0.05 probability level. In addition, regression analysis was used to discuss the relationship between root length and Suaeda growth indexes.

## 5. Conclusions

This study showed the interactions between salt and nitrogen on root development and ion uptake. The significant and higher ${\mathrm{Na}}^{+}$ and ${\mathrm{Cl}}^{-}$ uptake levels after the suitable N treatment suggested that N accelerated halophyte uptake of ions, thereby ameliorating the saline soil. The root biomass decreased along with N addition, which might be explained by a photosynthate allocation strategy that resulted in vital nutrient movement in shoots. The N addition distinctly promoted root elongation in surface layers at lower salt levels, whereas it increased root lengths in the subsoil to avoid salt injury during transpiration under higher-salinity conditions. Regression analysis confirmed that topsoil root elongation enhanced vegetative growth and ion uptake for halophyte. The results increased our knowledge of optimization and improvement in desalinization through N application-phytoremediation. Future experimental field work is essential to determine whether similar results will be achieved after N treatments of *Suaeda salsa*.

## Data Availability

The data will be provided upon request by the corresponding author.
